# Supplementary material for: Large scale discovery of coronavirus-host factor protein interaction motifs reveals SARS-CoV-2 specific mechanisms and vulnerabilities
Source: Nat Commun. 2021 Nov 19;12:6761. doi: 10.1038/s41467-021-26498-z (PMC8605023; doi:10.1038/s41467-021-26498-z)
Supplement: Supplementary file 1 — Supplementary Information [file 41467_2021_26498_MOESM1_ESM.pdf]

Supplementary information

Large scale discovery of coronavirus-host factor protein interaction motifs reveals SARS-CoV-2 specific mechanisms and vulnerabilities.

Kruse et al.

Content of supplementary information:

Supplementary Figure 1. FP affinity determinations.

Supplementary Figure 2. Network of coronavirus interactions with host factors

Supplementary Figure 3. Analysis of G3BP-N interaction

Supplementary Figure 4. Validation of ΦxFG motifs identified in ProP-PD

Supplementary Figure 5. Uncropped Western blot images

Table with primer sequences

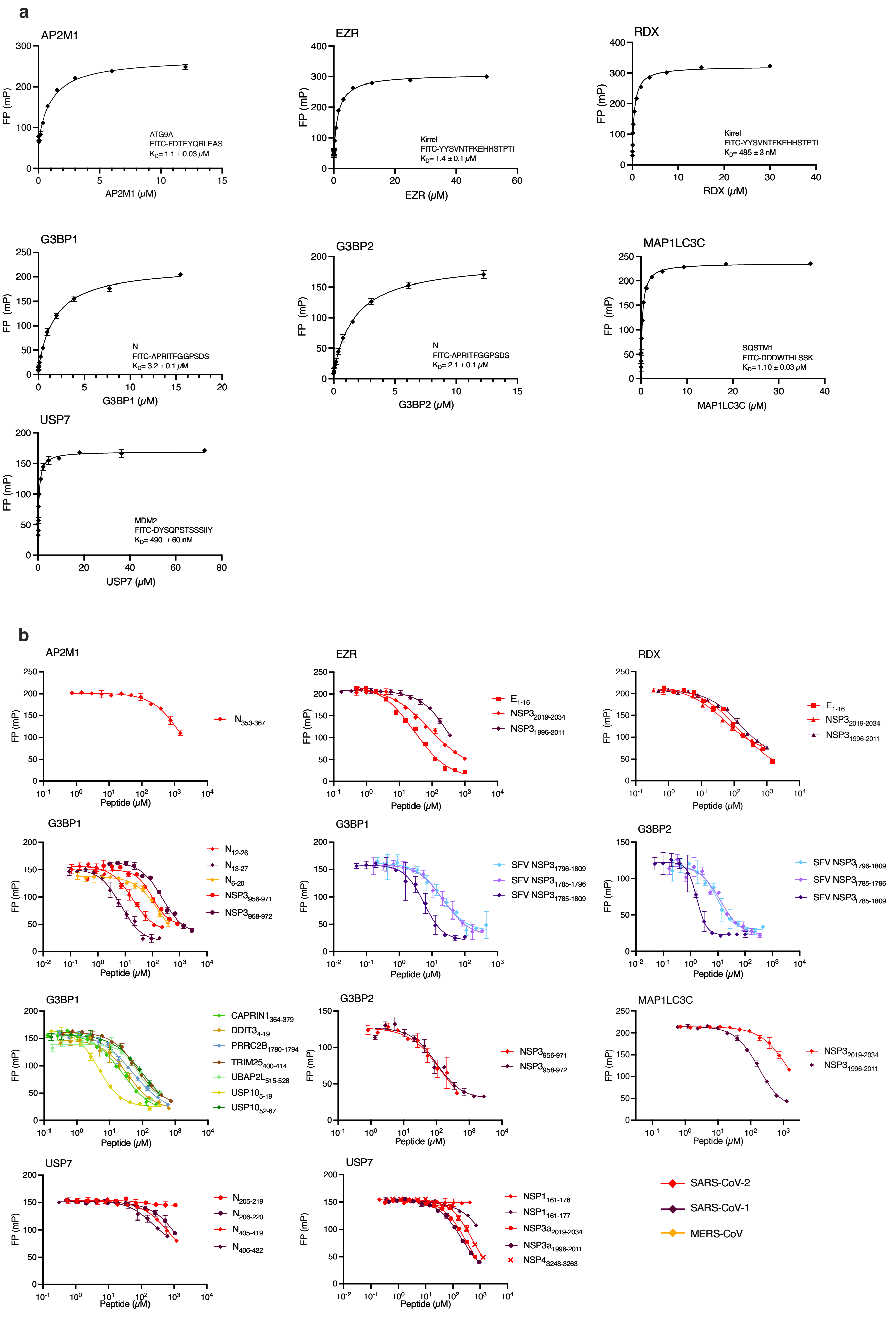

Supplementary Fig. 1. FP affinity determinations
a. Determination of KD values of the FITC-labelled probe peptides. Error bars expressed as mean ± SD. **b.** Affinity determinations through FP competition experiments using unlabeled peptides. All experiments were performed in triplicates.

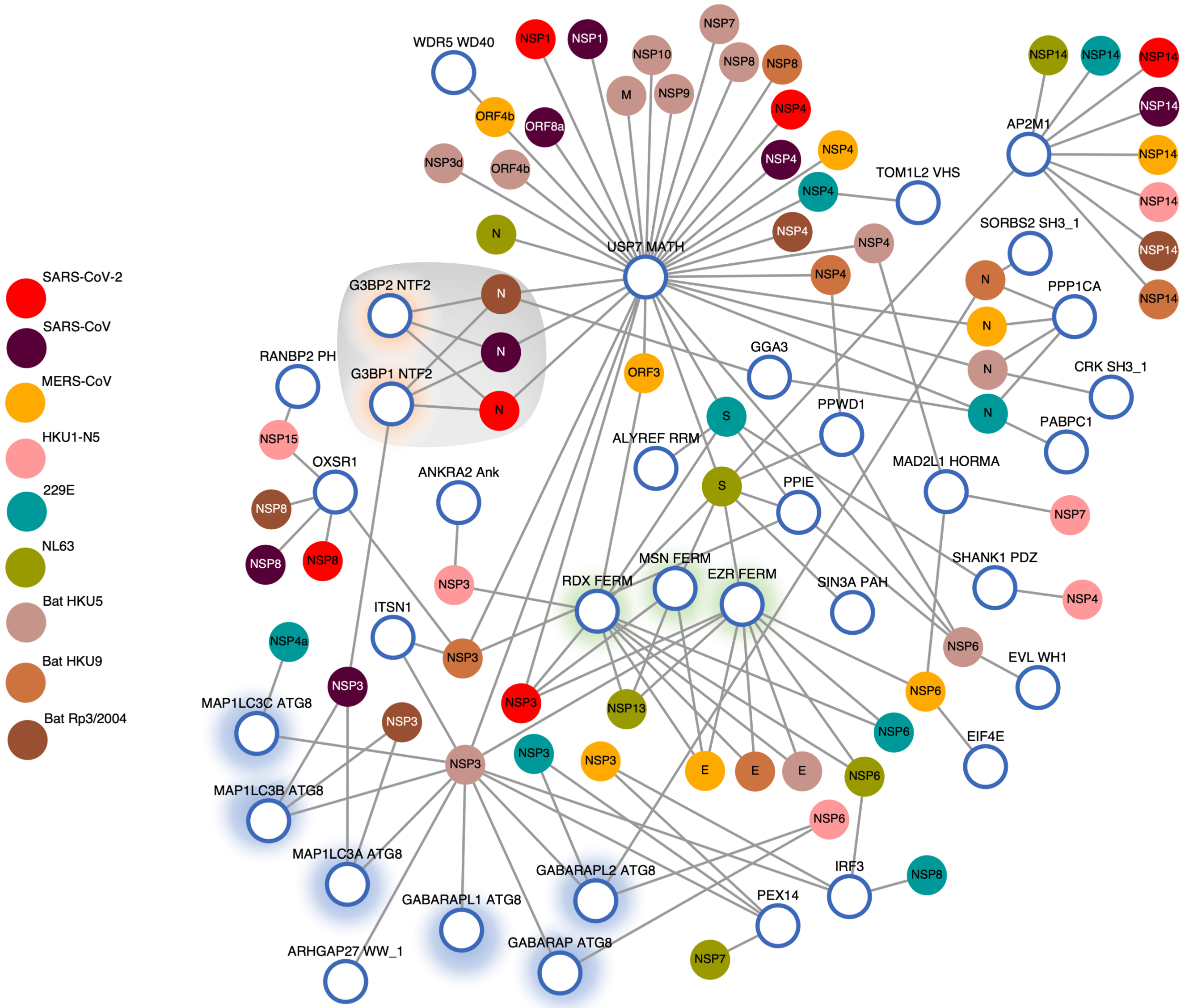

Supplementary Fig 2. Network of coronavirus interactions with host factors
Interactions between indicated coronavirus (human and bat) proteins and host factors based on the results from the ProP-PD screen.

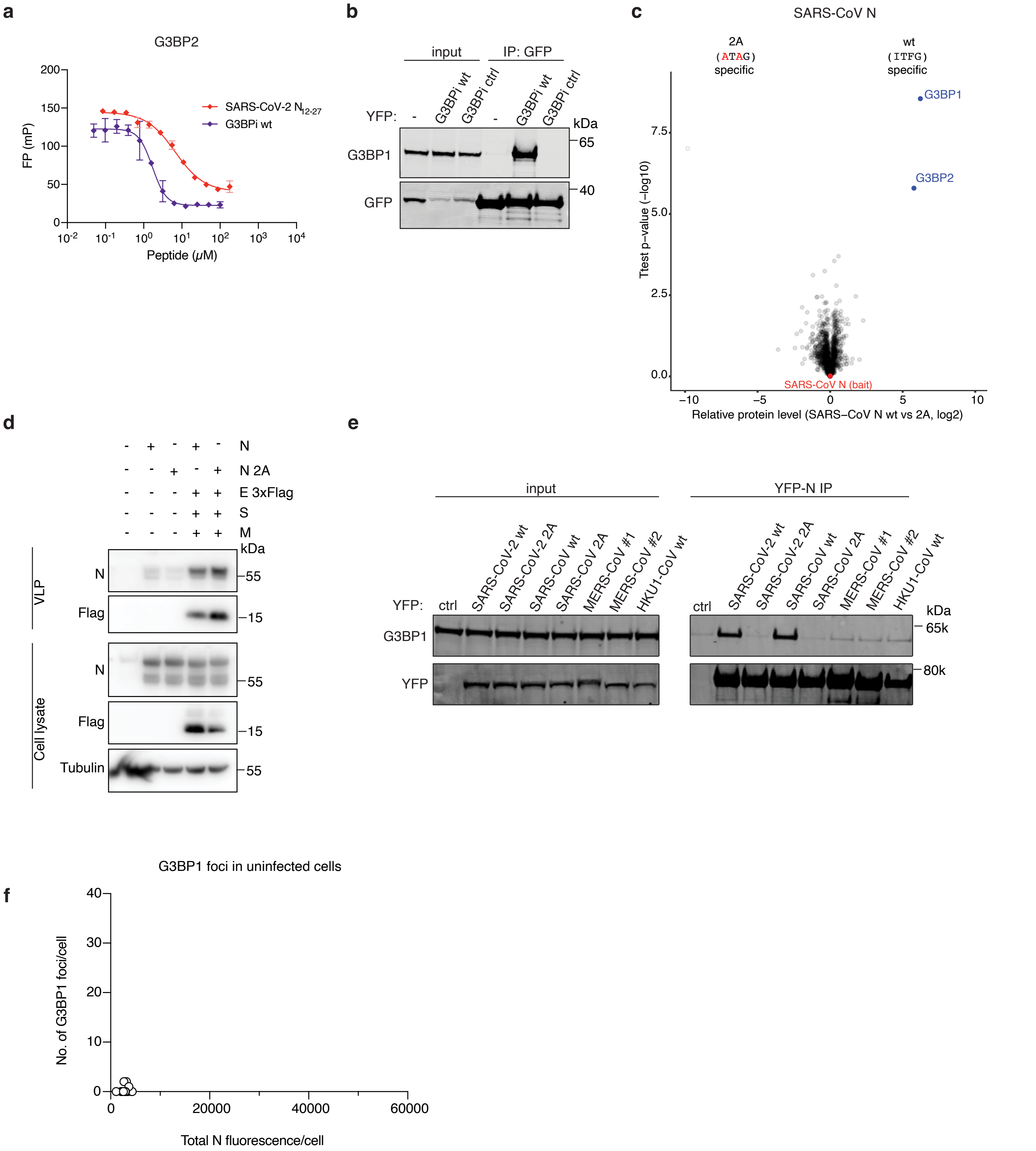

Supplementary Fig.3. Analysis of G3BP-N interaction

a. In vitro affinity measurements between recombinant G3BP2 NTF2 and the SFV nsP3 and SARS-CoV-2 N peptides as indicated (n= two biological duplicates each containing three technical replicates). Shown is a representative plot. Error bars expressed as mean ± SD. **b.** Purification of YFP tagged G3BPi wt and ctrl from HeLa cells and probed for binding to G3BP1 by Western blotting. Shown is a representative blot from two independent experiments. **c.** Quantitative mass spectrometry comparison of YFP tagged SARS-CoV N wt and 2A purified from HeLa cells. **d.** Virus assembly assay comparing the release of virus–like particles containing N wt and N 2A (n=2). **e.** YFP affinity purification of indicated N proteins from HeLa cells and probed for G3BP1 binding. Shown is a representative blot from three independent experiments. Two different strains of MERS (#1 and #2) were analysed due to differences in sequence at the N terminal region of N. **f.** Number of G3BP1 foci plotted against the total N fluorescence intensity per cell in mock infected cells. Data from one experiment done in duplicate with n=14 cells analysed. Source data are provided as a Source Data file for the data in f.

a

	K_D (µM)	SEM(µM)
$CAPRIN1_{364-379}$	174	4
$DDIT3_{4-19}$	14	4
$PRRC2B_{1780-1794}$	10	1
$TRIM25_{400-414}$	46	13
$UBAP2L_{515-528}$	34	3
$USP10_{5-19}$	0.52	0.24
$USP10_{52-67}$	40	5

b

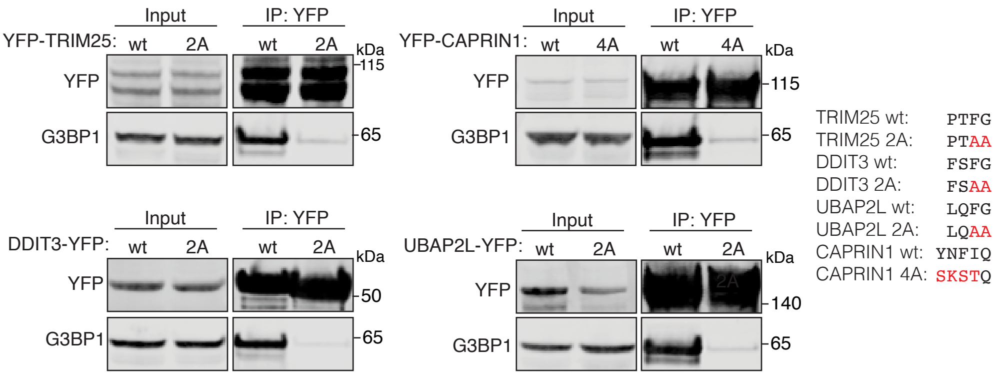

c

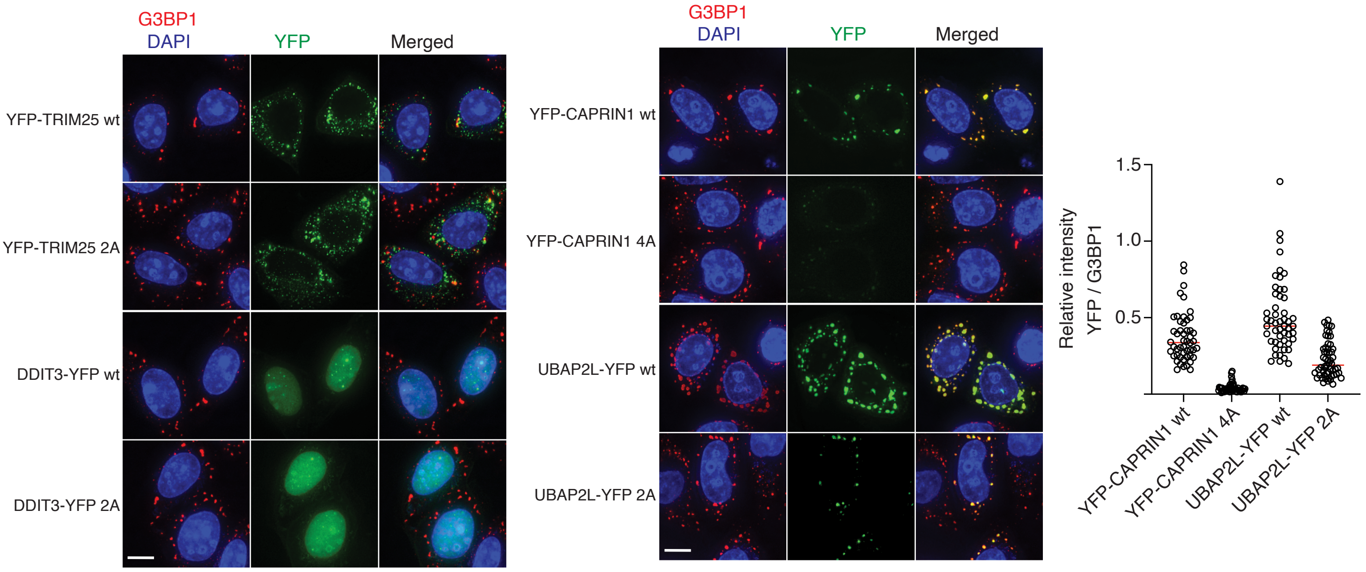

Supplementary Fig: 4: Validation of ΦxFG motifs in human proteins identified by ProP-PD

a. KD measurements of indicated peptides binding to G3BP2 (n=3). **b.** YFP affinity purification of indicated YFP tagged proteins either wild type (wt) or with mutated G3BP1 binding motif (2A or 4A) and probed for G3BP1 binding. Shown is a representative blot from two independent experiments. **c.** Analysis of the indicated proteins and their co-localisation with G3BP1 following arsenite treatment for 30 minutes by immunofluorescence microscopy. The co-localization of CAPRIN1 and UBAP2L with G3BP1 into foci was quantified. Red bar indicates median relative YFP/G3BP1 intensity, and each circle represents one co-localization event. At least five foci from 10 cells were measured. Microscopy images shown are representatives of three independent experiments. Scale bar is 5 µM. Source data are provided as Source Data file for for data in c.

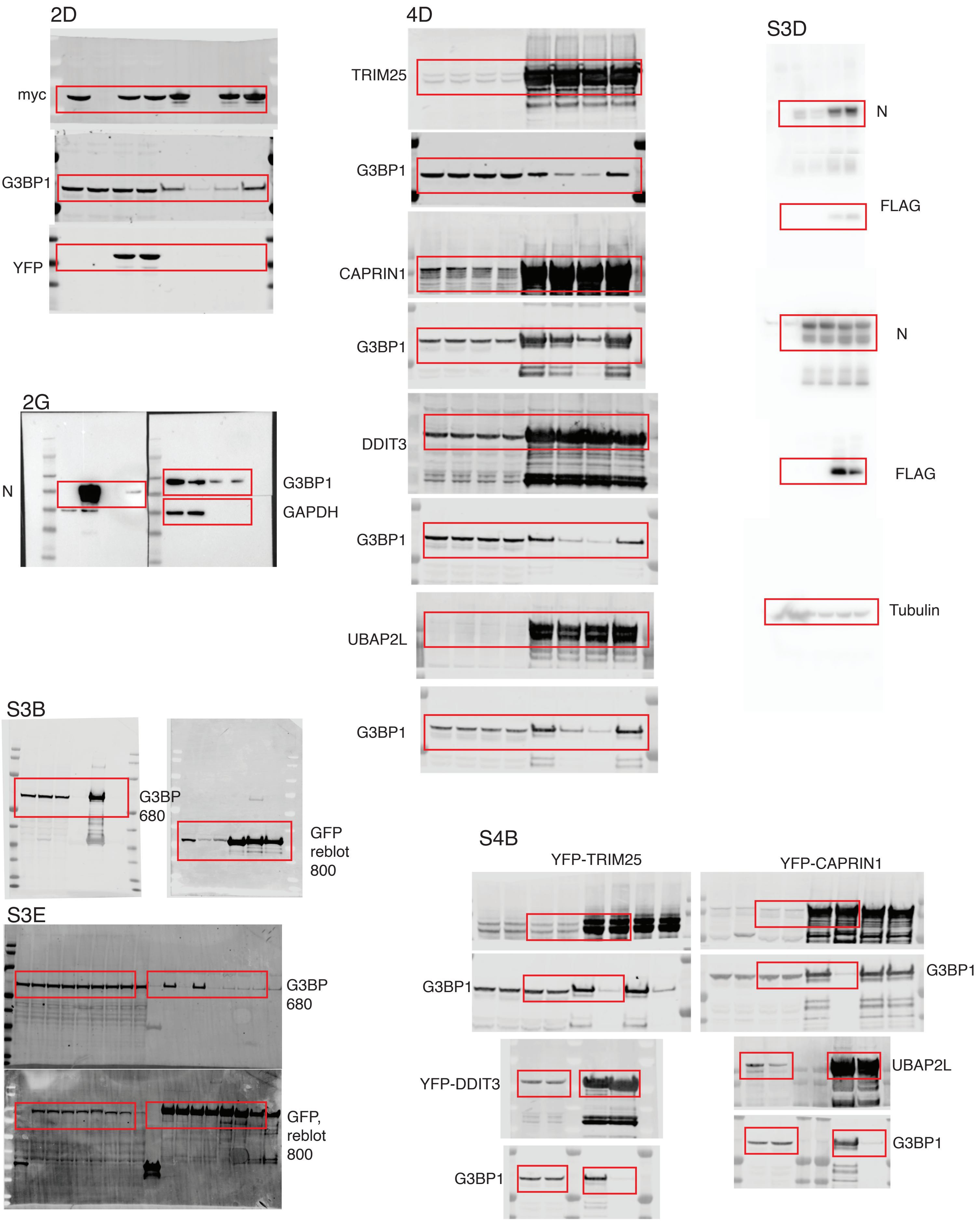

Supplementary Fig.5. Uncropped Western blot images

Primer name	Primer sequence
Trim25 2A fw	GCAAGCTTCCCACGGCTGCAGCCCCGGAACAGTTAGTGG
Trim25 2A rev	CCACTAACTGTTCCGGGGCTGCAGCCGTGGGAAGCTT
Caprin1 QC fw	GGCACAAATGCAGGGTCCCTCTAAGTCCACACAGGATTCAATGCTGG
Caprin1 QC rev	CCAGCATTGAATCCTGTGTGGACTTAGAGGGACCCTGCATTTGTGCC
UbaP2L- fw HindIII	GATCAAGCTTCCACCATGATGACATCGGTGGGCACTAACC
Uba-YFP rev	GATCGCGGCCGCGGGCTAGCGGAACCGTTGCCG
UbaP2L F518L F523G fw	GCTAAACCTGCAGTTAGGGGCATTGCAGGGTGGG TCAGAGCCTGTCC
UbaP2L F518L F523G re	GGACAGGCTCTGACCCACCCTGCAATGCCCCTAACTGCAGGTTTAGC
G3BP1-Venus fw	GATCAAGCTTCCACCATGGTGATGGAGAAGCCTAGTCC
G3BP1-Venus rev	GATCGCGGCCGCGGCTGCCGTGGCGCAAGCC
DDIT3 fw	GATCAAGCTTCCACCATGGCAGCTGAGTCATTGCC
DDIT3 rev	GATCGCGGCCGCGGTGCTTGGTGCAGATTCACCATTCG
DDIT3 qc fw	GTCATTGCCTTTCTCCGCCGCGACACTGTCCAGCTGG
DDIT3 qc rev	CCAGCTGGACAGTGTCGCGGCGGAGAAAGGCAATGAC
RiboVD1	5’ CAGCCTCTTCATCTGGC
RiboVD2	3’ GGTGGAGGATCCGGAG
SARS-CoV-2 qPCR fw	GTCATGTGTGGCGGTTCACT
SARS-CoV-2 qPCR rev	CAACACTATTAGCATAAGCAGTTGT
SARS-CoV-2 Probe	Fam-CAGGTGGAACCTCATCAGGAGATGC-BHQ
