# Supplementary material for: Large scale discovery of coronavirus-host factor protein interaction motifs reveals SARS-CoV-2 specific mechanisms and vulnerabilities
Source: Nat Commun. 2021 Nov 19;12:6761. doi: 10.1038/s41467-021-26498-z (PMC8605023; doi:10.1038/s41467-021-26498-z)
Supplement: Supplementary file 3 — Description of Additional Supplementary Files [file 41467_2021_26498_MOESM3_ESM.pdf]

**Description of Additional Supplementary Files**

File Name: Supplementary  Data 1
Description: **Table of expression constructs and proteins screened.** A complete list of protein domains including uniprot IDs and boundaries used for the screens.

File Name: Supplementary  Data 2
Description: **Table of viral ProP-PD results**. A list of peptides selected in the ProP-PD experiments including information on bait and strain for the interaction.

File Name: Supplementary  Data 3
Description: **Overview of measured affinities including peptide information**. Table providing detailed information on the affinities measured between viral peptides and protein domains.

File Name: Supplementary  Data 4
Description: **Mass spectrometry data.** Table with complete information of the mass spectrometry data.

File Name: Supplementary  Data 5
Description: **ProP-PD results for G3BP1/2 selections.** Table of peptides selected with G3BP1/2 as baits.
