# Supplementary material for: Large scale discovery of coronavirus-host factor protein interaction motifs reveals SARS-CoV-2 specific mechanisms and vulnerabilities
Source: Nat Commun. 2021 Nov 19;12:6761. doi: 10.1038/s41467-021-26498-z (PMC8605023; doi:10.1038/s41467-021-26498-z)
Supplement: Supplementary file 9 — Reporting Summary [file 41467_2021_26498_MOESM9_ESM.pdf]

nature portfolio

Corresponding author(s): Anna K Överby
Jakob Nilsson
Ylva Ivarsson

Last updated by author(s): Sep 24, 2021

Reporting Summary

Nature Portfolio wishes to improve the reproducibility of the work that we publish. This form provides structure for consistency and transparency in reporting. For further information on Nature Portfolio policies, see our Editorial Policies and the Editorial Policy Checklist.

Statistics

For all statistical analyses, confirm that the following items are present in the figure legend, table legend, main text, or Methods section.

n/a	Confirmed	
☐	☒	The exact sample size (n) for each experimental group/condition, given as a discrete number and unit of measurement
☐	☒	A statement on whether measurements were taken from distinct samples or whether the same sample was measured repeatedly
☐	☒	The statistical test(s) used AND whether they are one- or two-sided<br>*Only common tests should be described solely by name; describe more complex techniques in the Methods section.*
☒	☐	A description of all covariates tested
☒	☐	A description of any assumptions or corrections, such as tests of normality and adjustment for multiple comparisons
☐	☒	A full description of the statistical parameters including central tendency (e.g. means) or other basic estimates (e.g. regression coefficient) AND variation (e.g. standard deviation) or associated estimates of uncertainty (e.g. confidence intervals)
☐	☒	For null hypothesis testing, the test statistic (e.g. F, t, r) with confidence intervals, effect sizes, degrees of freedom and P value noted<br>*Give P values as exact values whenever suitable.*
☒	☐	For Bayesian analysis, information on the choice of priors and Markov chain Monte Carlo settings
☒	☐	For hierarchical and complex designs, identification of the appropriate level for tests and full reporting of outcomes
☒	☐	Estimates of effect sizes (e.g. Cohen's d, Pearson's r), indicating how they were calculated

Our web collection on statistics for biologists contains articles on many of the points above.

Software and code

Policy information about availability of computer code

Data collection	Mass spectrometric raw files were collected with MaxQuant (version 1.6.7.0) software (doi.org/10.1074/mcp.TIR119.001720)<br>For imaging on Deltavision Elite microscope: Softworx version 5.0.0 (Cytiva)<br>For imaging on Zeiss 710 confocal microscope: ZEISS ZEN Imaging Software version 14.0.19.201 (ZEISS)<br>ImageStudio version 3.1.4. (LI-COR)<br>Label-free quantification was performed with the MaxLFQ algorithm described in DOI: 10.1074/mcp.M113.031591<br>Leica Application Suit X software version 3.5.7.23225 (Leica)
Data analysis	Proteomics data analysis was performed with Perseus version 1.6.2.3 (doi:10.1038/nmeth.3901)<br>For analysis of next generation sequencing in-house custom Python scripts was used as described in (doi.org/10.1101/2021.04.13.439572)<br>For data analysis and graphing: Graphpad Prism version 9 (Graphpad software)<br>Cytoscape version 3.8.1<br>For imaging analysis of micrographs obtained with the Deltavision Elite microscope: Softworx version 5.0.0 (Cytiva)<br>Image analysis: ImageJ (fiji) version 1.52p.

For manuscripts utilizing custom algorithms or software that are central to the research but not yet described in published literature, software must be made available to editors and reviewers. We strongly encourage code deposition in a community repository (e.g. GitHub). See the Nature Portfolio guidelines for submitting code & software for further information.

Data

Policy information about availability of data

All manuscripts must include a data availability statement. This statement should provide the following information, where applicable:

- Accession codes, unique identifiers, or web links for publicly available datasets
- A description of any restrictions on data availability
- For clinical datasets or third party data, please ensure that the statement adheres to our policy

The mass spectrometry proteomics data have been deposited to the ProteomeXchange Consortium via the PRIDE partner repository (https://doi.org/10.1073/pnas.091062498) with the dataset identifier PXD025410: http://proteomecentral.proteomexchange.org/cgi/GetDataset?ID=PXD025410
A complete list of the viral strains in the library is available at http://slim.icr.ac.uk/phage_libraries/rna_viruses/species.html.
Transmembrane and the extracellular regions of transmembrane proteins were identified using UniProt (https://www.uniprot.org).
The details of the llibrary designs including the viral strains, proteins, peptides and statistics are available at http://slim.icr.ac.uk/phage_libraries/rna_viruses/species.html.
For peptide mapping and annotation, an RNA virus search database was added to the PepTools (http://slim.icr.ac.uk/tools/peptools/) web server.
The identity of proteins localizing to stress granules was retrieved from the HIPPIE database (http://cbdm-01.zdv.uni-mainz.de/~mschaefer/hippie/).
Disorder Predictions using IUPred (https://iupred3.elte.hu/).
Raw data is provided as source data for Fig 2A, 2E, 2F, 3C, 3E, Sup Fig 4C. Source data are provided with this paper.

Field-specific reporting

Please select the one below that is the best fit for your research. If you are not sure, read the appropriate sections before making your selection.

[x] Life sciences [ ] Behavioural & social sciences [ ] Ecological, evolutionary & environmental sciences

For a reference copy of the document with all sections, see nature.com/documents/nr-reporting-summary-flat.pdf

Life sciences study design

All studies must disclose on these points even when the disclosure is negative.

Sample size	No sample size calculation was performed using statistical methods. Sample sizes were chosen based on similar studies performed previously and typical ranges of effects observed for the types of assays used in this work. For infection experiments in Fig. 2A, E, F the experiments were repeated at least twice in triplicates and the data from the experiments is provided in source data. For mass spectrometry experiments the purifications were done in quadruplicates and all samples analysed in parallel.
Data exclusions	We excluded experiments where we could see that the controls did not work or where tissue culture plates were contaminated. No predetermined exclusion criteria was established.
Replication	Our experimental findings were reliably reproduced through repeated experiments and using different approaches. The number of biologically independent replicates and individual data points are indicated in the figure legends.
Randomization	We did not randomize our experiments. Randomization was not possible for the types of experiments included in this work.
Blinding	Quantification of G3BP1 foci in infected cells (Fig. 3E) was done automatically using Fiji software and therefore no blinding was required. For infection assays and biochemical assays all samples were treated in the same way, and therefore blinding is not relevant.

Reporting for specific materials, systems and methods

We require information from authors about some types of materials, experimental systems and methods used in many studies. Here, indicate whether each material, system or method listed is relevant to your study. If you are not sure if a list item applies to your research, read the appropriate section before selecting a response.

Materials & experimental systems

n/a	Involved in the study	
[ ]	[x]	Antibodies
[ ]	[x]	Eukaryotic cell lines
[x]	[ ]	Palaeontology and archaeology
[x]	[ ]	Animals and other organisms
[x]	[ ]	Human research participants
[x]	[ ]	Clinical data
[x]	[ ]	Dual use research of concern

Methods

n/a	Involved in the study	
[x]	[ ]	ChIP-seq
[x]	[ ]	Flow cytometry
[x]	[ ]	MRI-based neuroimaging

Antibodies

Antibodies used	Anti-mouse c-Myc (1:1000, Santa Cruz Biotechnology, sc-40), rabbit anti-G3BP1 (WB; 1:1000, Cell Signaling Technology, #17798S), mouse anti-G3BP1 (IF; 1:1000, Abcam, ab56574), GFP-Booster_Atto488 (IF 1:300, ChromoTek), mouse anti-GFP (WB, 1:1000, Roche, 11814460001), rabbit anti-GFP (WB; 1:5000, in-house), rabbit anti-SARS-CoV-2 nucleocapsid(WB 1:2500; IF 1:500; Sino Biological Inc., 40143-R001), mouse APC-conjugated antibody directed against dsRNA J2 (IF 1:200, Scicons, 10010500) mouse anti-3xFlag M2 (WB 1:25000, Sigma, F1804), rabbit anti-Tubulin (WB 1:4000, Abcam, ab6046), SARS-CoV-2 nucleoprotein (1:500; Invitrogen, #MA5-29981), G3BP1 (1:1000; SantaCruz Biotechnology, #sc-365338) and GAPDH (1:1000; SantaCruz Biotechnology, #sc-47724), donkey anti-rabbit IgG (Invitrogen, A32794), goat anti-rabbit HRP conjugated antibody (WB 1:2000 or 1:5000, Thermo Fisher Scientific, 31460), goat anti-mouse HRP conjugated antibody (WB 1:5000, Thermo Fisher Scientific, 31430), goat anti-mouse Alexa Fluor 546 (IF 1:1000, Invitrogen, A-11003), donkey anti-rabbit Alexa555 (IF 1:500, Thermo Fisher Scientific, a31572), donkey anti-mouse Alexa488 (IF 1:500, Thermo Fisher Scientific, a21202), goat IRDye 800CW anti-Mouse IgG (WB 1:10000, Li-Cor Biosciences, 926-32210), goat IRDye 800CW anti-Rabbit IgG (WB 1:10000, Li-Cor Biosciences, 926-32211), goat IRDye 680RD anti-Mouse IgG (WB 1:10000, Li-Cor Biosciences, 926-68070), goat IRDye 680RD anti-Rabbit IgG (WB 1:10000, Li-Cor Biosciences, 926-68071).
Validation	All antibodies have been validated by the manufacturer, in other publications or in-house using established protocols suitable for antibody validation. Specifically, for western blot and IPs a clear presence or absence of the western blot band at the predicted molecular weight was used to validate the antibody. For immunofluorescence, the presence or absence of the signal in the stained cells expressing or lacking the antigen were used for validation.<br>1) anti- mouse c-Myc (sc-40) This Myc antibody is recommended for detection of c-Myc p67 and c-Myc tagged fusion proteins of mouse, rat, human, monkey, feline and canine origin by WB, IP, IF, IHC(P), FCM and ELISA (https://www.scbt.com/p/c-myc-antibody-9e10)<br>2) rabbit anti-G3BP1 (Cell Signaling Technology, #17798S) Application Key: WB-Western Blot IP-Immunoprecipitation IHC-Immunohistochemistry ChIP-Chromatin Immunoprecipitation IF-Immunofluorescence F-Flow Cytometry E-P-ELISA-Peptide (https://www.cellsignal.com/products/primary-antibodies/g3bp1-antibody/17798)<br>3) mouse anti-G3BP1 (Abcam, ab56574) Suitable for: WB, IHC-P, Flow Cyt, ICC/IF (https://www.abcam.com/g3bp-antibody-2f3-ab56574.html)<br>4) GFP-Booster_Atto488 (ChromoTek) anti-GFP VHH/ Nanobody conjugated to a fluorescent dye for IF/ microscopy (https://www.chromotek.com/products/detail/product-detail/gfp-booster/)<br>5) mouse anti-GFP (WB, 1:1000, Roche, 11814460001) Application: Monoclonal antibody for detection of both wild-type and mutant forms of GFP or GFP fusions using: Immunoprecipitation, Western blots, Immunostaining (https://www.sigmaaldrich.com/DK/en/product/roche/11814460001)<br>6) rabbit anti-GFP (in-house) Validated by Western blotting by presence or absence of the western blot band at the predicted molecular weight of transfected and non-transfected cells.<br>7) rabbit anti-SARS-CoV-2 nucleocapsid (Sino Biological Inc., 40143-R001). Validated applications: WB,ELISA,IHC-P,ICC/IF (https://www.sinobiological.com/antibodies/cov-nucleocapsid-40143-r001)<br>8) mouse APC-conjugated antibody directed against dsRNA J2 (Scicons, 10010500) Uses: Immunofluorescence, immunehistology, immune electronmicroscopy, immunoblotting, immunprecipitation, ELISA (https://www.labome.com/product/SCICONS/10010500.html)<br>9) mouse anti-3xFlag M2 (Sigma, F1804) Application: For highly sensitive and specific detection of FLAG fusion proteins by immunoblotting, immunoprecipitation (IP) , immunohistochemisty, immunofluorescence and immunocyotchemistry. Optimized for single banded detection of FLAG fusion proteins in mammalian, plant, and bacterial expression systems (https://www.sigmaaldrich.com/DK/en/product/sigma/f1804).<br>10) rabbit anti-Tubulin (Abcam, ab6046) Suitable for: WB, ICC/IF, IHC-P, IP (https://www.abcam.com/beta-tubulin-antibody-loading-control-ab6046.html)<br>11) anti-rabbit SARS-CoV-2 nucleoprotein (Invitrogen, #MA5-29981), Applications: Western Blot (WB), Immunocytochemistry (ICC/IF), ELISA (https://www.thermofisher.com/antibody/product/SARS-SARS-Cov-2-Coronavirus-Nucleocapsid-Antibody-clone-5-Monoclonal/MA5-29981)<br>12) anti-mouse G3BP1 (SantaCruz Biotechnology, #sc-365338) G3BP1 Antibody (H-10) is recommended for detection of G3BP1 of human origin by WB, IP, IF, IHC(P) and ELISA (https://www.scbt.com/p/g3bp1-antibody-h-10)<br>13) anti-mouse GAPDH (SantaCruz Biotechnology, #sc-47724) GAPDH Antibody (0411) is recommended for detection of GAPDH of human origin by WB, IP, IF and IHC(P); not recommended for detection of GAPDH of mouse or rat origin (https://www.scbt.com/p/gapdh-antibody-0411)

Eukaryotic cell lines

Policy information about cell lines

Cell line source(s)	All cell lines were obtained from ATCC. The cell lines used are HeLa, HEK293,VeroE6 and Caco2.
Authentication	Parental cell lines were not authenticated.
Mycoplasma contamination	Our stocks of HeLa cells and the HEK293,VeroE6 and Caco2 cell lines were screened for mycoplasma contamination. No mycoplasma was detected.
Commonly misidentified lines (See ICLAC register)	We do not use cell lines that are commonly misidentified.
